# Prevalence of Low Plasma Vitamin B1 in the Stroke Population Admitted to Acute Inpatient Rehabilitation

**DOI:** 10.3390/nu12041034

**Published:** 2020-04-10

**Authors:** Reza Ehsanian, Sean Anderson, Byron Schneider, David Kennedy, Vartgez Mansourian

**Affiliations:** 1 Department of Physical Medicine and Rehabilitation, Vanderbilt University, Nashville, TN 37212, USA; rehsanian@salud.unm.edu (R.E.); sean.anderson@vumc.org (S.A.); byron.j.schneider@vumc.org (B.S.); david.j.kennedy@vumc.org (D.K.); 2 Department of Neurosurgery, Stanford University, Palo Alto, CA 34304, USA; 3 Division of Physical Medicine and Rehabilitation, Department of Neurosurgery, University of New Mexico School of Medicine, Albuquerque, NM 87131, USA

**Keywords:** thiamine, thiamin, vitamin B1, thiamine deficiency, stroke rehabilitation, acute rehabilitation humans

## Abstract

Objective: To determine the prevalence of vitamin B1 (VitB1) deficiency in the stroke population admitted to acute inpatient rehabilitation. Design: Retrospective cohort study. Setting: Acute inpatient rehabilitation facility at an academic medical center. Participants: 119 consecutive stroke patients admitted to stroke service from 1 January 2018 to 31 December 2018. Interventions: Not applicable. Main Outcome Measures: Plasma VitB1 level. Results: There were 17 patients (14%; 95% CI 9–22%) with low VitB1 with a range of 2–3 nmol/L, an additional 58 (49%; CI 40–58%) patients had normal low VitB1 with a range of 4–9 nmol/L, twenty-five patients (21%; CI 15–29%) had normal high VitB1 with a range of 10–15 nmol/L, and nineteen patients (16%; CI 10–24%) had high VitB1 with a range of 16–43 nmol/L. Conclusions: In this cohort of patients admitted to the stroke service at an acute rehabilitation facility, there is evidence of thiamine deficiency. Moreover, the data suggest that there is inadequate acute intake of VitB1. Given the role of thiamine deficiency in neurologic function, further study of the role of thiamine optimization in the acute stroke rehabilitation population is warranted.

## 1. Introduction

As coenzymes, the B vitamins play an important biochemical role in many enzymatic processes that are critical to cellular physiological functioning, including catabolic energy production as well as the anabolic production and processing of bioactive molecules [1]. The role of the B vitamins in metabolic function as well as their role in neurochemical synthesis makes them particularly important in the brain [1,2]. The importance of B vitamins in the brain is highlighted by the fact that they are transported by facilitated diffusion as well as via active transport across the blood–brain barrier and are stringently regulated by multiple homeostatic mechanisms [3,4]. In particular, vitamin B1 (VitB1), generally known as thiamin or thiamine, is a coenzyme critical to the synthesis of neurotransmitters essential for brain function [5]. VitB1 has been demonstrated to be a neuromodulator of acetylcholine, a function distinct from the action as a cofactor of metabolic processes [6]. Indeed, VitB1 has great significance in proper brain function, not only because of its role as a coenzyme important in carbohydrate metabolism, but also due to its contribution to the structure and function of neuronal and neuroglia cell membranes, another function distinct from its role in metabolism [7,8]. The ubiquitous role of VitB1 in the brain explains the fast turnover rates in the brain, spinal cord, and peripheral nervous system [9,10].

The health consequences of VitB1 deficiency are commonly thought of as the symptoms and signs of the disease beriberi, including a cardiac form with risk of high output heart failure with associated edema, and a neurological form with chronic peripheral neuropathy with more advanced symptoms manifesting as Wernicke’s encephalopathy and Wernicke-Korsakoff Syndrome [11,12,13,14,15,16]. Although classically categorized as “wet”, “dry”, “childhood”, and “infantile”, more modern classification recognizes that the symptoms and signs may or may not be associated with edema and vary according to the age of the patient and the presence of other vitamin deficiencies (11–13). The diagnosis of inadequate VitB1 status is classically a clinical diagnosis and, if left untreated, may lead to devastating and irreversible neurological damage.

It is important to note that, even with biochemical evidence of VitB1 deficiency, associated clinical signs and symptoms may not present as they lack sensitivity and specificity [17,18]. Moreover, the diagnostic criteria are nonspecific and the diagnosis may be missed in many patients [14,19,20]. Given that the signs and symptoms of VitB1 inadequacy are vague and common to many different disorders [21], identifying patients with risk factors, such as alcoholism, persistent emesis, malnutrition, intestinal malabsorption, and viral infection, has been the key to focusing the clinician to properly identify VitB1 inadequacy [14,22,23,24,25]. The identification of specific patient groups at higher risk of inadequate VitB1 status, including patients with systemic inflammation and severe sepsis, with trauma and burns, with congestive heart failure, with renal failure, post-cardiac surgery, and after prolonged inadequate nutritional intake, have improved identification and remediation of VitB1 inadequacy [17,25]. Identifying if patients admitted to the acute inpatient rehabilitation setting post-acute stroke are at higher risk of VitB1 inadequacy is important given the role of VitB1 in brain function and the potential role in recovery post-stroke.

Given the role of VitB1 deficiency in brain function and the clinical utility of identifying susceptible patient groups in diagnosing VitB1 inadequacy, the present study aims to describe the plasma levels of VitB1 in a cohort of patients admitted to an acute inpatient rehabilitation hospital after an acute stroke.

## 2. Methods

### 2.1. Patient Population

This is an Institutional-Review-Board-approved, retrospective cross-sectional study that analyzed the results of routine measurements of plasma VitB1 of 119 consecutive patients with a diagnosis of stroke admitted to stroke service from 1 January 2018 to 31 December 2018, at Vanderbilt Stallworth Rehabilitation Hospital. Given the minimal risk and retrospective nature of the study, the Institutional Review Board approved a waiver of informed consent. All patient demographic history, medical history, and laboratory values were extracted from the electronic medical record utilized at the Rehabilitation Hospital and combined with the laboratory results stored in the electronic medical record of the acute hospital at Vanderbilt University Medical Center. Any general rehabilitation patients who were admitted to the stroke service for primary diagnosis other than stroke were omitted from this review, leaving a sample of a total 119 stroke patients.

### 2.2. VitB1 Measurement

Plasma VitB1 measurements were performed at admission to the stroke acute rehabilitation service with high-performance liquid chromatography (HPLC)-based upon the method previously described [26]. The test has been developed and characteristics determined by ARUP Laboratories (500 Chipeta Way, SLC, UT 84108; 1-800-522-2787), see compliance statement (aruplab.com/cs). Unpublished results were provided by the ARUP Institute for Clinical and Experimental Pathology from a reference internal study as part of a method development and validation in November 2007. Samples were collected and tested from 59 female and 57 male (116 total participants) adults (>18 years old), self-reported to be healthy adults living in Utah, age 19–64 years (mean 36 years), not taking vitamins. The reference interval for vitamin B1, measured in plasma as unphosphorylated thiamine, was 4–15 nmol/L. The range compares well to older published values 9.5 +/− 3.3 nmol/L listed in Tietz Clinical Guide to Laboratory Tests, 3rd edition, 1995. Therefore, established reference range of normality for plasma VitB1 for this study and used clinically in our acute rehabilitation facility ranged from 4 to 15 nmol/L and is used as the laboratory reference standard.

The values used in the analysis were those utilized in determining the clinical care of patients on the stroke acute inpatient service. Patients were grouped as follows according to their plasma VitB1 levels: Low VitB1 (<4 nmol/L), Normal Low VitB1 (4–9 nmol/L), Normal High VitB1 (10–15 nmol/L), High VitB1 (>15 nmol/L).

### 2.3. Statistical Analysis

The statistical analysis was conducted using GraphPad Prism (version 8.0.1 for Windows, GraphPad Software, La Jolla California, CA, USA).

Descriptive statistics included the calculation of the 95% confidence interval of the median, mean with standard deviation and standard error, the coefficient of variation, skewness, and kurtosis for values in the four VitB1 groupings. Differences between low, normal low, normal high, and high groups were analyzed using the Brown-Forsythe ANOVA test for continuous data and the Chi-square test of independence or Fisher’s exact test for categorical variables. A *p* value of < 0.05 was considered as the limit of significance.

## 3. Results

### 3.1. Patient Characteristics

The 17 patients in the low plasma group had an average age of 58.18 years (SD = 17.72); the 58 patients in the normal low group had an average age of 61.93 years (SD = 15.62); the 25 patients in the normal high group had an average age of 66.20 years (SD = 13.84); and the 19 patients in the high group had an average age of 63.37 years (SD = 15.53). The Brown-Forsythe ANOVA test showed no statistically significant difference across groups, F (3, 69.65) = 0.926, *p* = 0.433. There was also no statistically significant difference for Body Mass Index (BMI) across groups, F (3, 46.23) = 0.778, *p* = 0.512 (Table 1).

A Chi-square test of independence was performed to examine the relation between gender and plasma thiamine levels. The relation between these variables was not significant, X^2^ (3, *N* = 119) = 0.58, *p* = 0.901. A Chi-square test of independence was also performed to examine the relationship between ethnicity and plasma thiamine levels displaying no significant difference, X^2^ (3, *N* = 119) = 1.33, *p* = 0.722 (Table 1).

The Chi-squared approximation requires the expected frequency of every cell in the contingency table to be greater than 5. Given that one or more of the expected frequencies in the analysis for the type of stroke is less than 5, the chi-square approximation may be incorrect, and therefore a Fisher’s exact test was performed, which did not reveal a significant difference between groups (two-sided Fisher’s exact test *p* = 0.620) (Table 1).

Given the impact of different comorbidities on the VitB1 levels, major comorbidities for individuals in each group was extracted from the medical record and hypertension was the most frequent comorbidity across all groups (Table 1). The Chi-square test of independence for major comorbidities also did not reveal a significant difference, X^2^ (12, *N* = 119) = 9.881, *p* = 0.626 (Table 1).

### 3.2. Plasma Thiamine Levels

#### 3.2.1. Low VitB1

**Figure 1:**
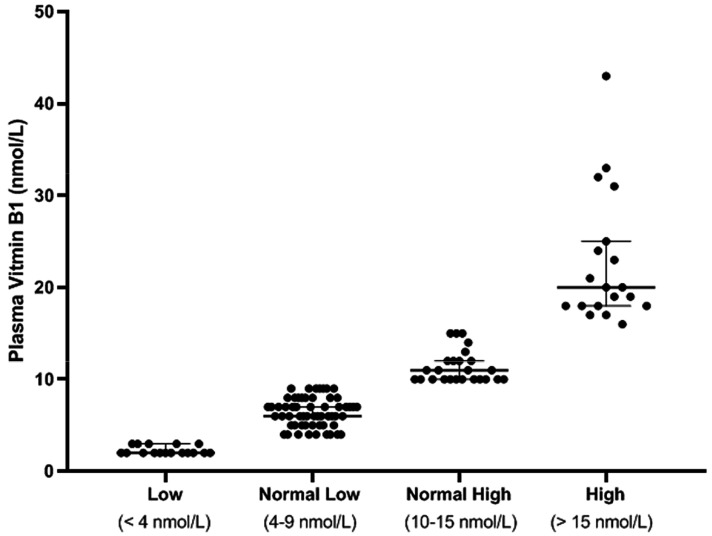
Plasma vitamin B1 levels. The patient cohort is divided into four groups with the normal range divided equally into normal low and normal high.

There were 17 (14%; 95% CI 9–22%) patients with low VitB1 with a range of 2–3 nmol/L, 95% CI of median actual confidence level of 95.10% lower confidence limit (LCL) 2, upper confidence limit (UCL) 3, mean 2.29 (SD 47, SE 0.11), coefficient of variation 20.47%, Skewness.994, Kurtosis −1.17 (Table 2 and Figure 1).

#### 3.2.2. Normal Low VitB1

There were 58 (49%; CI 40–58%) patients with normal low VitB1 with a range of 4–9 nmol/L, 95% CI of median actual confidence level of 95.21% with LCL 6 and UCL 7, mean 6.41 (SD 1.58, SE 0.21), coefficient of variation 24.61%, Skewness.026, Kurtosis −0.96 (Table 2 and Figure 1).

#### 3.2.3. Normal High VitB1

There were 25 (21%; CI 15–29%)patients with normal high VitB1 with a range of 10–15 nmol/L, 95% CI of median actual confidence level of 95.67% with LCL 10 and UCL 12, mean 11.36 (SD 1.75, SE 0.351), coefficient of variation 15.43%, Skewness 1.06, Kurtosis −0.122 (Table 2 and Figure 1).

#### 3.2.4. High VitB1

There were 19 (16%; CI 10–24%) patient with high vitamin B1 with a range of 16–43, 95% CI of median actual confidence level of 98.08% with LCL 18 and UCL 25, mean 22.74 (SD 7.16, SE 1.64), coefficient of variation 31.51%, Skewness 1.61, Kurtosis 2.28 (Table 2 and Figure 1).

## 4. Discussion

This retrospective cohort demonstrates that patients admitted to an acute rehabilitation hospital, after an acute stroke, have low plasma levels of VitB1 (14%; 95% CI 9–22%) and low normal VitB1 levels (49%; CI 40–58%). Combined, this represented 63% of patients with VitB1 inadequacy (low or low normal levels of VitB1). As a retrospective cohort, this study is unable to determine causality, specifically if patients with inadequate VitB1 are susceptible to stroke or if patients admitted to an acute care hospital with stroke are at risk for quickly developing low plasma levels of VitB1.

The finding of such a significant number of low normal patients must be taken into account with the understanding of what defines the “normal” range of VitB1. The current established VitB1 range is normalized based on healthy populations. The concept of differential VitB1 levels and requirements for those in a pathological state has been proposed in light of dementia and delirium studies [27]. The current reference ranges may not be valid in a post-stroke patient cohort, and the authors agree with the proposal that current reference data for thiamine deficiency should be called into question as they may not accurately describe VitB1 inadequacy in a pathological state as they were based on normalized values in healthy populations [27]. Given the increasing literature describing the importance of VitB1 in brain function among not only patients suffering from alcoholism [28,29] but also the geriatric population suffering from cognitive deficits [30], critically ill patients [17,31,32], post-bariatric procedures [33], and those suffering from conditions such as Alzhemers [34], HIV/AIDS [35], malignancy [36], diabetes [37], and obesity [5,38], it is important to consider the potential role of low and low normal levels of VitB1 in the population recovering from acute stroke.

### 4.1. VitB1 Deficiency Mimicking Stroke

The importance of identifying VitB1 deficiency in patients with stroke is highlighted by the fact that this metabolic deficiency may mimic acute stroke. VitB1 deficiency may present with a sudden neurological deficit, which is indistinguishable from acute onset stroke. Swenson has reported the CT findings in thiamine deficiency-induced coma [39]. It has been proposed that CT perfusion scan can be utilized to differentiate stroke, comma and VitB1 deficiency; however, the diagnosis could also be made simply with high dose IV VitB1 administration after laboratory draw to confirm VitB1 inadequacy. Indeed, the radiological presentation of hypoattenuation [39] and intraventricular hemorrhage [40,41], along with the similar clinical signs and symptoms of VitB1 inadequacy, makes this metabolic disturbance prone to misdiagnosis. This similarity may lead to the improper diagnosis of stroke, especially in patients with a prior diagnosis of vascular pathology, and lead to improper treatment, as reported by Karapanayiotides et al. [42]. This group reported a case study of a patient who responded well to treatment with high-dose VitB1 and complex B vitamins and recovered fully three months after her initial presentation. The importance of identifying patient groups, such as those post-bariatric surgery when considering VitB1 inadequacy versus stroke, was highlighted by Blum et al. [43]. They identified a post-bariatric surgery who was misdiagnosed with transient ischemic attack. The misdiagnosis of ischemic stroke may have been precipitated by mimicking symptoms, as the patient presented to Blum et al. displaying left-sided motor weakness with sensory deficit and paresthesias and numbness in the left arm and leg; however, without any CT lesions, they properly suspected post-gastrectomy Wernicke’s encephalopathy and IV administration of VitB1 immediately resolved the patients symptoms [43]. It should be noted that stroke should remain on the differential after bariatric surgery, as Cho et al. reported generalized seizures and stroke with brain infarction on CT, post-gastric bypass [44].

### 4.2. VitB1 and Stroke

Aside from being a potential stroke mimic, diagnosing VitB1 inadequacy in patients pre- and post-stroke has many potential implications. If low and/or low normal VitB1 levels are a risk factor for stroke, this may have significant public health implications. Alternatively, if patients are developing VitB1 inadequacy during their continuum of care for stroke, this may have implications on early nutritional need in this setting. Regardless of causality, there are potential implications for the acute rehabilitation setting. Defining and identifying this association is the first step in further investigating if the repletion of VitB1 in these patients during their continuum of care may play a role in optimizing their recovery. This latter point may be most important given the increased metabolic demands of the brain at baseline and especially post-stroke [45], similar to the increased metabolic demand seen in the VitB1 deficiency of chronic alcoholics [46]. The increased metabolic demand post-stroke was observed as early as the 1970s by investigators reporting an increase of up to 100% in energy requirements of patients with hemiparetic gait [47,48]. This increased metabolic demand, compounded by the risk of poor nutritional intake [49], may represent a multi-factorial relationship explaining the findings of this study.

### 4.3. Plasma VitB1

Traditionally, it has been thought that the most accurate measurement of VitB1 status is attained by measuring the “TDP effect”, which consists of measuring transketolase enzyme activity in erythrocyte hemolysates in the presence and absence of thiamin diphosphate [50]. However, it has been established that direct measurement via HPLC has equivalent precision to the erythrocyte transketolase activation assay and both yield similar results [26,51].

Plasma VitB1 levels can be checked with relative ease, and changes in thiamine in whole blood are not significantly different to changes in plasma [52] after VitB1 supplementation. Although measurements of blood VitB1 levels have been traditionally criticized as having low sensitivity and specificity, the HPLC method for assessing thiamin diphosphate has been shown to be equivalent to the erythrocyte activation assay [51] and there have been a number of direct HPLC methods developed to measure VitB1 in plasma, erythrocytes, whole blood, and urine [26,51,53,54,55,56]. Conditions which typically lead to transient changes in VitB1 in blood measurements, such as acute trauma [17], sepsis [17,57], coronary artery bypass graft surgery [58], bariatric surgery [59,60], renal failure [61], diuresis with diuretic [12,50], uncontrolled diabetes [62], or acute hepatic injury [63], were either not present or not significantly different between the low, normal low, normal high, and high subgroups. Moreover, although using plasma VitB1 levels to assess VitB1 status has been classically cautioned against, as it accounts for less than 10% of total blood VitB1 [51], studies have found that plasma concentration of VitB1 correlates well with erythrocyte thiamine diphosphate [52,64] and is the most responsive measure to oral VitB1 intake [64]. Plasma VitB1 levels have been shown to respond to oral supplementation with VitB1 [52,65,66], and therefore this measurement is of keen interest in the rehabilitation of patients post-stroke, as they may not have access to IV replenishment as they progress through the continuum of rehabilitation care.

### 4.4. Study Limitations

There are potential limitations, as the study is retrospective and observational in design. Although this retrospective cohort demonstrates that there is a subset of patients admitted to acute inpatient rehabilitation after an acute stroke that has VitB1 inadequacy, causality cannot be assessed. Furthermore, both the reference values and data set reported are not from a random sample and are therefore prone to inherent bias; consecutive patient enrollment as well as a strict adherence to inclusion/exclusion criteria and study duration were applied in order to limit this bias. This study is also limited in its ability to assess for clinical differences, if any exist, between VitB1 subgroups. Moreover, this study is unable to assess the role of VitB1 repletion during acute inpatient rehabilitation. Another potential limitation stems from the fact that plasma and serum thiamine levels may be prone to inaccuracy when determining total body VitB1 levels, as they have been shown to be decreased in settings such as the Intensive Care Unit (ICU) with critically ill trauma patients and those requiring continuous renal replacement therapy [17,23]. However, given that this patient cohort was under different metabolic demands than those that are critically ill and did not include those with end-stage renal disease, the use of plasma VitB1 may be appropriate. Whole blood assays may be a better reflection of total body stores but are impractical in an acute care setting due to cost and/or long turnaround time. Furthermore, thiamine deficiency may occur within 9–18 days of inadequate intake [67,68,69,70,71] but rapidly recover with supplementation, and, given that plasma VitB1 is accurate in determining recent intake, it may be more useful in tracking the appropriate supplementation of patients admitted for acute rehabilitation post-stroke. In addition, the patient population represents a single acute rehabilitation hospital and results may differ in a center with differing patient demographics, further limiting the generalizability of our results. Moreover, the lack of dietary data is another major limitation of the study. Specific dietary intake was not controlled and/or specifically documented in the electronic medical record, which may lead to further discrepancies in the data. However, it is assumed that the majority of patients adhered to the prescribed diet approved by the hospital nutritional staff, and, if the prescribed diet was supplemented by patients, it is assumed that it did not vary between groups.

## 5. Conclusions

This retrospective cohort demonstrates that there is a subset of patients admitted to an acute rehabilitation hospital for rehabilitation after an acute stroke with low plasma VitB1 (14%; 95% CI 9–22%) with another subset of patients that has low normal VitB1 levels (49%; CI 40–58%). These findings warrant further investigation given the integral function of VitB1 in neuronal function and the potential for VitB1 deficiency to impair neuronal function and recovery

## Figures and Tables

**Table 1:** Patient characteristics.

	Low	Normal Low	Normal High	High	*p*
**N**	17	58	25	19	
**Age (years)**	58.18 (17.72)	61.29 (15.62)	66.2 (13.84)	63.37 (15.53)	0.433
**BMI**	30.23 (11.66)	28.52 (7.25)	26.25 (5.08)	27.9 (8.33)	0.512
**Gender**					
Male	9	33	12	10	
Female	8	25	13	9	0.901
**Ethnicity**					
White	11	40	19	15	
Non-White	6	18	6	4	0.722
**Stroke Type**					
Hemorrhagic	2	12	8	7	
Ischemic	13	40	15	11	
Both	2	6	2	1	0.620
**Comorbidities**					
Hypertension	11	48	20	16	
Hyperlipidemia	8	28	13	7	
Cardiac Dysfunction	4	19	4	6	
Renal Dysfunction	2	14	4	9	
Diabetes Mellitus	9	18	8	7	0.626

The patient cohort is divided into four groups with the normal range divided equally into normal low and normal high. The number of patients, average age in years with standard deviation in parenthesis, average BMI with standard deviation in parenthesis, Gender, Ethnicity, Stroke Type, and Comorbidities are shown. For continuous variables, Brown-Forsythe ANOVA revealed no significant difference between groups. For categorical variables the Chi-square test of independence was performed, unless this calculation was not valid, in which case Fisher’s exact test was performed. There was no significant difference between groups.

**Table 2:** Plasma vitamin B1 levels.

	Low	Normal Low	Normal High	High
**Number of values**	17	58	25	19
**Minimum**	2	4	10	16
**25% Percentile**	2	5	10	18
**Median**	2	6	11	20
**75% Percentile**	3	8	12	25
**Maximum**	3	9	15	43
**95% CI of median**				
Actual confidence level	95.10%	95.21%	95.67%	98.08%
Lower confidence limit	2	6	10	18
Upper confidence limit	3	7	12	25
**Mean**	2.294	6.414	11.36	22.74
**Std. Deviation**	0.4697	1.579	1.753	7.164
**Std. Error of Mean**	0.1139	0.2073	0.3506	1.643
**Coefficient of variation**	20.47%	24.61%	15.43%	31.51%
**Skewness**	0.9936	0.02553	1.161	1.607
**Kurtosis**	−1.166	−0.9582	0.1225	2.277

The patient cohort is divided into four groups, with the normal range divided equally into normal low and normal high. Descriptive statistics of the plasma level of thiamine (nmol/L) are presented.
